# Cannabis-Induced Hypodopaminergic Anhedonia and Cognitive Decline in Humans: Embracing Putative Induction of Dopamine Homeostasis

**DOI:** 10.3389/fpsyt.2021.623403

**Published:** 2021-03-30

**Authors:** Kenneth Blum, Jag Khalsa, Jean Lud Cadet, David Baron, Abdalla Bowirrat, Brent Boyett, Lisa Lott, Raymond Brewer, Marjorie Gondré-Lewis, Gregory Bunt, Shan Kazmi, Mark S. Gold

**Affiliations:** ^1^Western University Health Sciences, Pomona, CA, United States; ^2^Institute of Psychology, ELTE Eötvös Loránd University, Budapest, Hungary; ^3^Division of Nutrigenomics, Precision Translational Medicine, LLC., San Antonio, TX, United States; ^4^Division of Nutrigenomics, Genomic Testing Center, Geneus Health, LLC., San Antonio, TX, United States; ^5^Department of Psychiatry, University of Vermont, Burlington, VT, United States; ^6^Department of Psychiatry, Wright University Boonshoff School of Medicine, Dayton, OH, United States; ^7^Department of Microbiology, Immunology and Tropical Medicine, George Washington University, School of Medicine, Washington, DC, United States; ^8^Molecular Neuropsychiatry Research Branch, DHHS/NIH/NIDA Intramural Research Program, National Institutes of Health, Baltimore, MD, United States; ^9^Department of Neuroscience, Interdisciplinary Center (IDC), Herzliya, Israel; ^10^Bradford Health Services, Madison, AL, United States; ^11^Department of Psychiatry and Behavioral Sciences, Howard University College of Medicine, Washington, DC, United States; ^12^Good Samaritan/Day Top Treatment Center, and NYU School of Medicine, New York, NY, United States; ^13^College of Osteopathic Medicine of the Pacific, Western University of Health Sciences, Pomona, CA, United States; ^14^Department of Psychiatry, Washington University School of Medicine, St Louis, MO, United States

**Keywords:** cannabis use disorder, depression, anhedonia, neuroanatomic alterations, reward deficiency syndrome, genetic testing, pro-dopamine regulation, dopamine homeostasis

## Abstract

Over years, the regular use of cannabis has substantially increased among young adults, as indicated by the rise in cannabis use disorder (CUD), with an estimated prevalence of 8. 3% in the United States. Research shows that exposure to cannabis is associated with hypodopaminergic anhedonia (depression), cognitive decline, poor memory, inattention, impaired learning performance, reduced dopamine brain response-associated emotionality, and increased addiction severity in young adults. The addiction medicine community is increasing concern because of the high content of delta-9-tetrahydrocannabinol (THC) currently found in oral and vaping cannabis products, the cognitive effects of cannabis may become more pronounced in young adults who use these cannabis products. Preliminary research suggests that it is possible to induce 'dopamine homeostasis,' that is, restore dopamine function with dopamine upregulation with the proposed compound and normalize behavior in chronic cannabis users with cannabis-induced hypodopaminergic anhedonia (depression) and cognitive decline. This psychological, neurobiological, anatomical, genetic, and epigenetic research also could provide evidence to use for the development of an appropriate policy regarding the decriminalization of cannabis for recreational use.

## Prevalence

Cannabisis regarded as the most abused illicit drug in the world today. An estimated 150–200 million people use cannabis regularly, and a relatively common disorder, known as cannabis use disorder (CUD), has an estimated prevalence of 8.3% in young adults in the United States (1, 2). A recent survey of 482 young college students, ~19–20 years, found that 29% of students vaped cannabis. From this survey, men from high socioeconomic status (SES) vaped higher cannabis amounts than men 13–14 years from lower SES status and women (3). Between 2000 and 2016, the lifetime and daily use of cannabis among 12th graders was 44 and 6%, respectively. In 2019, 8th graders' ~13–14 years, past-year use was 11.8%, and past-month use was 6.6%, 28.8% of 10th graders had used marijuana in the past year and 18.4% in the past month. Among 12th graders, ~17–18 years, rates of cannabis use grew to 35.7% during the previous year and 22.3% in the previous month. Reports of daily and near-daily use were 6.4%. Almost 4% of 12th grader teens vape cannabis products daily (NIH What is the scope of marijuana use in the United States? Marijuana Research Report, https://www.drugabuse.gov/ [accessed October 28, 2020]).

More importantly, there is increasing concern by the addiction medicine community that because of the high content of delta-9-tetrahydrocannabinol (Δ9-THC), (the chemical that causes the high) currently found in edibles and vaping cannabis vaping products [up to 90%; https://www.marijuanabreak.com/90-percent-thc-weed, (accessed January 20, 2020)], the chronic cannabis users may develop more severe hypodopaminergic-anhedonia (depression) and cognitive decline. Incidentally, other serious respiratory and pulmonary consequences, including chronic obstructive pulmonary disorder (COPD), have also been reported among those who use e-vaping devices (4).

## Cannabis and Neuroanatomic Alterations and Cognition

Cannabidiol (CBD) can ameliorate the effects of THC and protect the brain from damages, possibly through CB1 antagonism (5). These psychophysiological damages include dose-dependent psychotic cognitive and behavioral symptoms (6) and observed from several human structural neuroimaging studies frequency of use dependent reductions in gray matter volumes. The reductions occur in the medial temporal cortex, orbitofrontal cortex, temporal poles, parahippocampal gyrus, and insula. Chronic cannabis users also display significant neuroanatomic alterations in the medial temporal, frontal cortex, cerebellum (7), and the fusiform gyrus, temporal pole, superior temporal gyrus, and occipital cortex (8).

A top area of concern, especially in young developing adults, is the damaging effect of high doses of Δ9-THC and consequent cognitive impairment. According to Floresco et al. (9) and Lorenzetti et al. (8), the neuroanatomic alterations in the prefrontal-hippocampal function and subsequent down-regulation of CB1 receptors may result in cognitive decline/working memory, decision-making, and inhibitory control in chronic cannabis users. Cannabinoid type 1 receptors (CB1) associated with motivational, emotional, and affective processing (10) are usually abundant in these areas, so upregulation of CBD1 receptors may positively affect THC-induced brain damage. Notably, these cognitive effects may return to normal after 4–6 weeks of abstinence from cannabis (11, 12).

## The Synaptic Mechanisms Underlying THC-Induced Anhedonia and Cognitive Deficits

In adult cannabis users, brain activation decreases in the middle temporal gyrus, insula, and striate area and increases in the superior and posterior transverse temporal and inferior frontal gyri and middle temporal gyrus. While activation in adolescents increases in the inferior parietal gyrus and putamen compared to healthy controls (13). Research suggests that functional alterations in these areas are neuroadaptive changes in cannabis users and may be compensatory (13).

## Cannabis and Dopaminergic Function

Chronic cannabis usage, including in adolescents, has also significantly reduced striatal dopamine release causing (hypodopaminergia) and associated poor memory, inattention, and impaired learning performance (14). Chronic use of cannabis observed with [18F]-DOPA PET found reduced brain dopamine synthesis and subsequently attenuated reward sensitivity, motivation, and induced apathy. It is noteworthy that the 9/9 allele polymorphism carriers have high D2/D3 receptor availability (due to higher dopamine re-absorption rates) compared to carriers of the 10/10 alleles in early-onset heavy cannabis users (15). The carriers of the 7R DRD4 polymorphism are likely to experiment with cannabis more than the non-carriers. According to Volkow et al. (16), among cannabis users, there is a reduced dopamine brain response linked to the emotionality and severity of the addiction. Cannabis users also show inversely correlated dopamine reactivity with higher negative emotionality scores relative to controls (17). There is some evidence that suggests large doses of Δ9-THC increase dopamine release by inhibiting VTA GABAergic activity (18). This effect may translate to an increased fear reaction in cannabis users. In animal experiments (19), the repeated administration of Δ9-THC induced depressive-like symptoms, including prolonged anhedonia due to CB1 type receptors' impairment and dopaminergic alterations in the mesolimbic region. This Δ9-THC induced dysfunction in animals associates with attenuated anandamide signaling. Interestingly, the subjects with CUD diagnosis and no baseline depressive symptoms were at the follow-up, four times more likely (age-adjusted) to have depressive symptoms than those with no CUD diagnosis (20).

In the past, the chronic use of cannabis of low potency (2–4% Δ9-THC) did not associate with significant neuroanatomic alterations, psychosis, or even depression. However, as the mean Δ9-THC concentration has increased substantially over the last 10 years, from 8.9 to 17.1% by 2017 (21), the use of cannabis products such as pastes, gummies, and e-vaping devices with still higher concentrations of Δ9-THC, reported as high as 90%, may result in a higher degree of hypodopaminergia, associated poor memory, inattention, and impaired learning performance in chronic cannabis users, especially among adolescents with cannabis use disorder. Thus, the brain changes and symptomatology that signify chronicity depend on potency and duration, frequency of use; smoking cannabis daily multiple times per day.

## Balancing Dopamine Function With Precision Pro-Dopamine Regulation

The functional neuroimaging techniques, such as resting-state functional magnetic resonance imaging (rsfMRI), have shown that acute exposure to cannabis reduces the neuronal activity in the nucleus accumbens (NAc) and prefrontal cortex (PFC), anterior cingulate gyrus (ACG), striatum, and thalamus. In contrast, chronic cannabis exposure increases the rsfMRI in these brain regions, and in adolescents' chronic use of high Δ9-THC content cannabis results in impaired motivation with depression, anhedonia, low academic achievement, and reduced functional connectivity in the brain reward circuitry (22, 23). The primary neurochemical insult is an altered dopaminergic function across mesolimbic pathways requiring neurotransmitter balance across the brain reward system. Nestor et al. (24) found that in chronic cannabis users (with an average of 6.1 [range = 2.5–17] lifetime years of cannabis use and with the consumption of 7,258-lifetime cannabis joints), there is an increased ventral striatal (VS) blood-oxygen-level-dependent (BOLD) response to stimuli predicted potential non-drug rewards. Importantly, VS hyperactivity is seen during reward anticipation associated with years of cannabis use and the lifetime estimation of numbers of cannabis joints consumed. Another known impairment related to chronic cannabis use relates to compulsive drug use with NMDA receptor-dependent synaptic depression located at the ventral tegmental area (VTA) linked to dopamine circuitry. Chronic cannabis exposure also activates VTA cannabinoid CB1 receptors and reduces transient neurotransmission at VTA local Glu-DA synapses by activating NMDA receptors and subsequent endocytosis of AMPA receptor GluR2 subunits (25).

**Figure 1:**
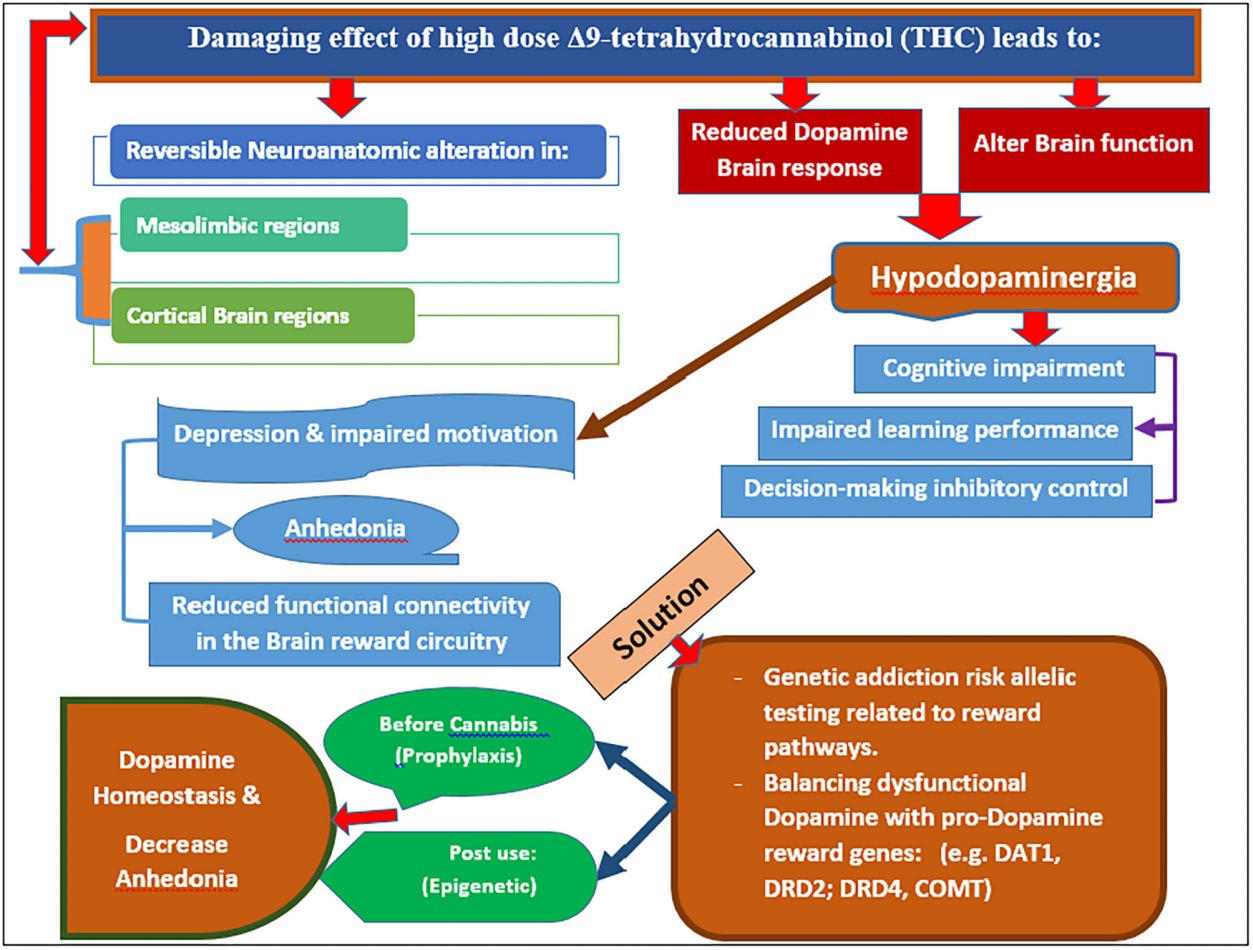
It is a Model proposed for combatting chronic use of potent cannabis and anhedonia (Original figure Blum 2020). Note: Potency of cannabis may be as high as 90% THC in gummies and vaping products.

This evidence provides possible new targets in obviating chronic addiction learning, specifically with chronic cannabis use in humans. Dopamine augmentation is difficult to achieve, especially after the development of a substance use disorder (SUD). Vigorous physical exercise, like Eminem, TMS, and nutraceuticals, have been proposed as viable options. Our proposal herein of incorporating genetic risk allelic testing related to reward pathways along with potential induction of dopamine homeostasis seems logical. This concept takes on even more importance when we consider that the onset and peak use of cannabis occur during brain development in teenagers and, as such, represents an unwanted window of liability (26, 27). The onset of cannabis use begins in the mid-teens and peaks by the age of 25, with the development of cannabis use disorder between 15 and 20. In order to either prevent or treat the high dose Δ9-THC-induced hypodopaminergic anhedonia and cognitive decline, it may be possible to combine the non-invasive testing for the genetic addiction risk score (GARS) with pro-dopamine regulation and restore the dopamine function (26–58). A novel model (Figure 1) espouses a reasonable biphasic approach; a short-term blockade followed by long-term dopaminergic upregulation with KB220Z^*^ primarily for reward deficiency syndrome (RDS) behaviors (29–38).

“^*^KB220Z ***Components, The most recent variant of KB220Z (powdered form),*** *is composed of the following ingredients: Vitamin B6, 10 mg (500%); Thiamine, 15 mg (1,033% of Daily Value); and Chromium poly nicotinate, 200 mcg (166%). A fixed-dose of synaptose is included as well, which is a combination of amino acids and herbs that contains DL-Phenylalanine, L-Tyrosine, Passion-Flower Extract; a Complex containing Arabinogalactans, N-Acetylglucosamine, Astragalus, Aloe Vera, Frankincense Resin, White Pine Bark Extract, and Spirulina; Rhodiola; L-Glutamine; 5-Hydroxytryptophan (5-HTP); Thiamine Hydrochloride; Pyroxidal-5-phosphate and Pyridoxine HCl, CoQ10, NADH, and N-Acetyl Cysteine (NAC);* (59). *The powder was manufactured by Cephram, Inc. (New Jersey)”*.

However, in chronic cannabis-using adolescents, the goal would be to enhance brain reward functional connectivity [measures the degree of synchrony of the BOLD time-series between different brain regions] and connectivity volume [Voxel-based morphology (VBM)], attenuate depression-like symptoms (anhedonia), and target stress-like anti-reward drug dependence symptoms. Using fMRI of both naïve animals (60) and heroin abstinent subjects (61), we confirmed blood-oxygen-level-dependent (BOLD) activation of dopaminergic reward pathways and recruitment of dopamine neuronal firing with KB220Z. These types of fMRI results provide some evidence for dopaminergic activation.

Millions of individuals worldwide struggle to combat their frustrating and even fatal romance with getting high daily. The neuroscience community conducts and funds incredible research using sophisticated molecular-genetic applied technology in animal experiments and humans using neuroimaging to advance our understanding of brain reward circuitry's complex functions that play a vital role in the expressed symptoms found in addictions. Although dopamine is known as a major neurotransmitter involved in addictions, many disagree about how to deal with dopamine dysregulation clinically to prevent and treat addictive disorders, including cannabis use disorder (CUD). An alternative approach could include two phases; a brief blockade followed by stable dopaminergic upregulation. The treatment goal would be to augment brain reward functional connectivity volume by targeting reward deficiency and the stress-like anti reward symptomatology of addiction. These phenotypes can be characterized using the Genetic Addiction Risk Score (GARS). Dopamine homeostasis may thus be achieved via “Precision Addiction Management” (PAM)®, the customization of neuronutrient supplementation based on the GARS test result, along with a behavioral intervention (29).

Dopaminergic homeostasis could be achieved by genetic testing for addiction risk and administering precursor amino acid and enkephalinase inhibitory, non-addictive, natural complex pro-dopamine regulator (KB220), matching to one's neurotransmitter pathways associated reward gene polymorphisms, as previously proposed. Fried et al. (59) reported a case series about the novel treatment of screening with GARS and utilizing a customized pro-dopamine regulator matched to polymorphic reward genes with a hypodopaminergic risk. The proband was a female of 34 years with a history of cannabis abuse and alcoholism. She voluntarily entered treatment after experiencing a car accident while driving under the influence. Following an assessment, she was genotyped using the GARS and given a polymorphic matched neuro-nutrient with a KB220Z base. She successfully recovered from Substance Use Disorder (SUD) and experienced improved socialization, family, economic status, well-being, and attenuation of major depression. She tested urine negative over the first 2 months in treatment and a recent screening. Following ~2 months into the program, her parents also decided to take the GARS and started taking the recommended variants. The proband's father (a binge drinker) and mother (no SUD) showed improvement in various behaviors. Finally, the proband's biological children were also GARS tested, showing a high risk for SUD. This three-generation case series represents an example of the impact of genetic information coupled with an appropriate DNA guided “Pro-Dopamine Regulator” to recover and enhance life.

Over the many years of the development of the putative pro-dopamine regulator, with the research ID code of KB220Z, there have been a plethora of studies showing remarkable benefits related to reward deficiency behaviors and associated drug and non-drug phenotypes (26, 27, 29, 51, 55, 56, 59, 60, 62–93).

**Figure 2:**
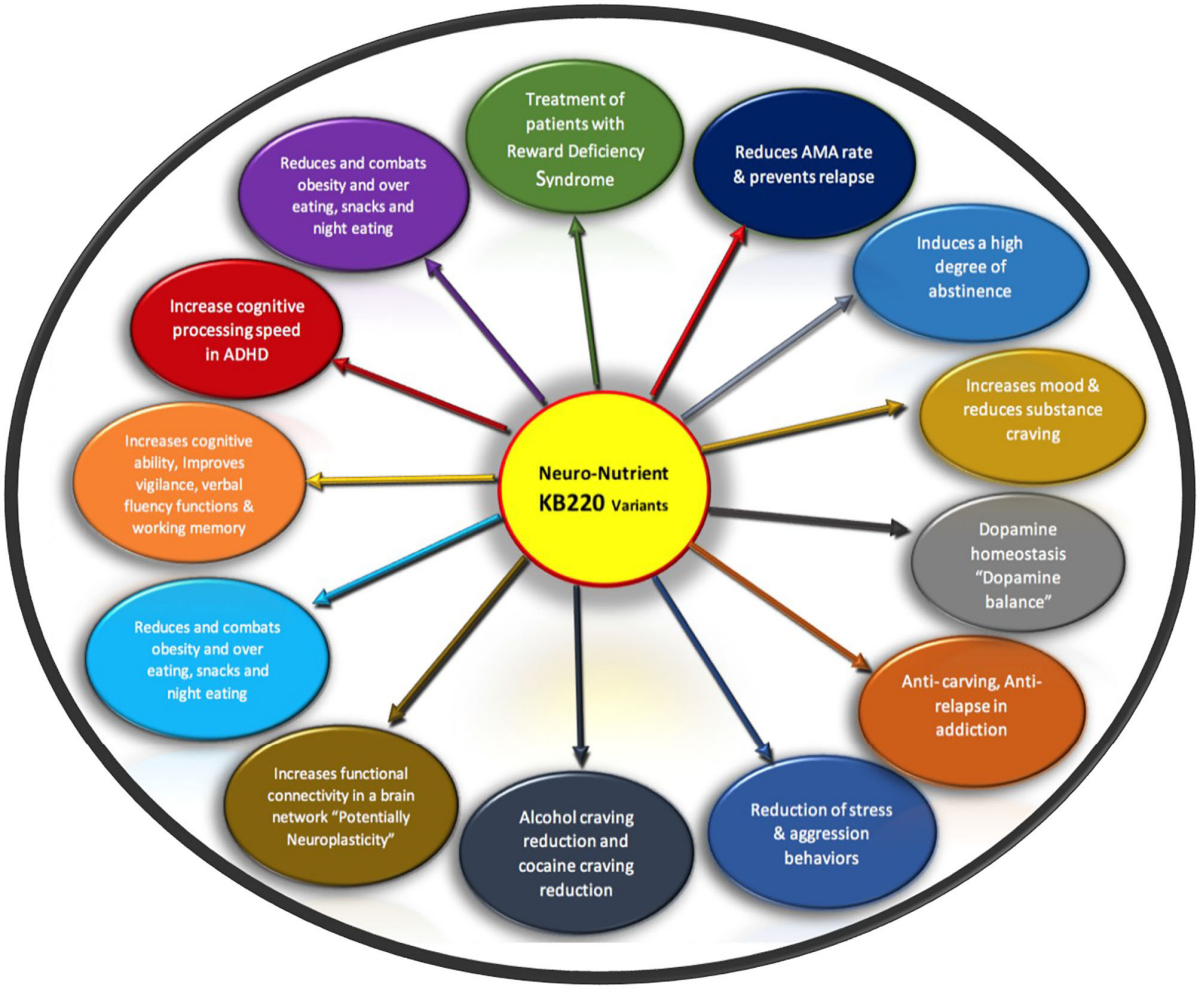
It is a schematic showing list of RDS benefits of KB220 variants Original figure Blum 2020.

This KB220Z variant has been the subject of at least 43 clinical and pre-clinical studies showing anti-RDS addictive behaviors via dopaminergic mechanisms [see Annotated Bibliography and review by Blum et al. (30)] and Figure 2.

Manza et al. (94) suggest that chronic cannabis abuse is associated with resting-state brain function changes, particularly in dopaminergic nuclei implicated in psychosis, habit formation, and reward processing. Is it possible that by utilizing GARS-guided precision, KB220Z could help restore the normal functioning of reward processing and connectivity in cannabis-using subjects, especially in our youth and high-risk populations?

## Issues of Cannabis Legalization

Even though extensive research shows that chronic use of cannabis is associated with significant adverse health effects (16, 95–97), there is a disturbing trend of many states in the United States (US), and other countries legalize cannabis for unregulated recreational and medicinal use. Colorado was one of the first two U.S. states to legalize cannabis for recreational use for adults 21 and older. There are serious concerns regarding physical and mental health risks, particularly among adolescents who may use cannabis of high THC content. According to Parnes et al. (98), two hypotheses have been studied. First, cannabis use among college students 21 years old and older would increase after recreational legalization. Second, there would be a positive correlation between the new cannabis legislation and out-of-state students' decision to attend a Colorado university as well as their cannabis use after that. However, the opposite was found. Data from a survey of 5,241 undergraduate students showed that cannabis use increased since recreational legalization for all students, particularly for those over 21 years. For past-month use frequency, no differences were found between pre-legalization and post-legalization (98).

Moreover, out-of-state students reported higher past 30-day use than in-state students. Indeed, one real concern relates to the post-legalization opening of retail cannabis stores and adult cannabis use throughout the country. Specifically, Everson et al. (99) evaluated this issue in Washington and found that frequent cannabis use grew significantly between 2009 and 2016 with greater access to cannabis retailers. Frequent use increased among adults living within 0.8 miles of a retailer. Moreover, Klimer (100) developed a 14-point policy as follows: (1) Production, (2) Profit motive, (3) Power to regulate, (4) Promotion, (5) Prevention and treatment, (6) Policing and enforcement, (7) Penalties, (8) Prior criminal records, (9) Product types, (10) Potency, (11) Purity, (12) Price, (13) Preferences for licenses, and (14) Permanency. A crucial aspect of moving forward in terms of legalization must address the high content of THC in waxes and other products, as well as statewide inconsistencies (101).

On the other hand, the American Society of Addiction Medicine (ASAM) issued a policy statement (102) on marijuana and cannabinoids, recommending *decriminalization* instead of legalization of cannabis and cannabinoids. Furthermore, the legalization of the commercial sale and promotion of cannabis with high THC content for recreational use in many states (Alaska, California, Colorado, Illinois, Maine, Massachusetts, Michigan, Nevada, Oregon, Vermont, and Washington) may lead to significant increases THC intoxication, dependence, and addiction because of the euphoria. Consequently, the neurochemical impact on reward systems in the brain that can lead to neurological reward system deficits may also be significant and of great concern to clinicians.

Thus, until an FDA-approved therapy for treating cannabis use disorder and any of its adverse health components, developing a safe and responsible strategy toward decriminalizing cannabis and cannabis products seems paramount in the United States. As such, consideration of using KB220Z, a dopamine up-regulator discussed above, for restoring balanced neurotransmission and alleviating hypodopaminergia and its consequences like anhedonia (depression), cognitive decline, and other mental health effects due to chronic cannabis use. Similarly, the supplement N-acetylcysteine (NAC) to treat substance use disorders, including CUD, could be useful. In a double-blind, randomized control trial of a cohort of cannabis-dependent adolescents, Gray et al. (103) demonstrated that NAC is an effective treatment for cannabis use disorder, and Tomko et al. (104) revied NAC as a potential treatment for substance use disorders, including cannabis.

## Conclusion

Although the prevalence of recreational cannabis users at high risk for developing anhedonia and depression is unknown, the amount of cannabis used (dose of THC) seems to be an important factor. Chronic use of high THC content cannabis, either by oral ingestion or vaping, results in reversible neuroanatomic alterations in the mesolimbic and cortical brain regions with subsequent hypodopaminergia and associated depression/anhedonia. Cannabis use among young adults causes these neuroanatomical and psychological changes, magnified by DNA polymorphisms in pro-dopamine reward genes (like DAT1, DRD2, DRD4, COMT). These DNA polymorphisms can be measured either before cannabis use (prophylaxis) or post-use (epigenetic). Treatment should involve the induction of dopamine homeostasis via pro-dopamine regulation and thereby ameliorate anhedonia. No FDA-approved therapies are currently available to treat CUD or any comorbidities, such as depression or cognitive decline (23, 58, 71, 94, 105–112). Using a dopamine up-regulator such as KB220Z to restore brain dopamine in hypodopaminergia until an FDA-approved therapy is available could be considered for chronic cannabis users with CUD. The development of an appropriate policy regarding the legalization of cannabis and cannabis products and decriminalization is needed.

## Author Contributions

AB developed the schematic. The original manuscript was developed by KB and JK, and all authors commented and equally contributed.

## Conflict of Interest

KB is the inventor and patent holder of both GARS and Pro-dopamine regulators. He has licensed same to Ivitalize Inc. KB owns stock in Ivitalize Inc. LL is a paid consultant fron Geneus Health, LLC. The remaining authors declare that the research was conducted in the absence of any commercial or financial relationships that could be construed as a potential conflict of interest.

## References

[1] Haberstick BC Young SE Zeiger JS Lessem JM Hewitt JK Hopfer CJ. Prevalence and correlates of alcohol and cannabis use disorders in the United States: results from the national longitudinal study of adolescent health. Drug Alcohol Depend. (2014) 136:158–61. 10.1016/j.drugalcdep.2013.11.022 24440049 PMC3963405

[2] Wu LT Zhu H Swartz MS. Trends in cannabis use disorders among racial/ethnic population groups in the United States. Drug Alcohol Depend. (2016) 165:181–90. 10.1016/j.drugalcdep.2016.06.002 27317045 PMC4939114

[3] Jones CB Hill ML Pardini DA Meier MH. Prevalence and correlates of vaping cannabis in a sample of young adults. Psychol Addict Behav. (2016) 30:915–21. 10.1037/adb0000217 27631612

[4] Ghosh A Coakley RC Mascenik T Rowell TR Davis ES Rogers K. Chronic e-cigarette exposure alters the human bronchial epithelial proteome. Am J Respir Crit Care Med. (2018) 198:67–76. 10.1164/rccm.201710-2033OC 29481290 PMC6034122

[5] Thomas A Baillie G Phillips A Razdan R Ross R Pertwee R. Cannabidiol displays unexpectedly high potency as an antagonist of CB1 and CB2 receptor agonists *in vitro*. Br J Pharmacol. (2007) 150:613–23. 10.1038/sj.bjp.0707133 17245363 PMC2189767

[6] Murray RM Englund A Abi-Dargham A Lewis DA Di Forti M Davies C. Cannabis-associated psychosis: Neural substrate and clinical impact. Neuropharmacology. (2017) 124:89–104. 10.1016/j.neuropharm.2017.06.018 28634109

[7] Batalla A Bhattacharyya S Yuce M Fusar-Poli P Crippa JA Nogue S. Structural and functional imaging studies in chronic cannabis users: a systematic review of adolescent and adult findings. PLoS ONE. (2013) 8:e55821. 10.1371/journal.pone.0055821 23390554 PMC3563634

[8] Lorenzetti V Solowij N Yüce M. The role of cannabinoids in neuroanatomic alterations in cannabis users. Biol Psychiatry. (2015) 79:e17–3. 10.1016/j.biopsych.2015.11.013 26858212

[9] Floresco SB Zhang Y Enomoto T. Neural circuits subserving behavioral flexibility and their relevance to schizophrenia. Behav Brain Res. (2009) 204:396–409. 10.1016/j.bbr.2008.12.001 19110006

[10] Battistella G Fornari E Annoni J-M Chtioui H Dao K Fabritius M. Long-term effects of cannabis on brain structure. Neuropsychopharmacology. (2014) 39:2041–8. 10.1038/npp.2014.67 24633558 PMC4104335

[11] Schreiner AM Dunn ME. Residual effects of cannabis use on neurocognitive performance after prolonged abstinence: a meta-analysis. Exp Clin Psychopharmacol. (2012) 20:420–9. 10.1037/a0029117 22731735

[12] Hirvonen J Goodwin RS Li CT Terry GE Zoghbi SS Morse C. Reversible and regionally selective downregulation of brain cannabinoid CB1 receptors in chronic daily cannabis smokers. Mol Psychiatry. (2012) 17:642–9. 10.1038/mp.2011.82 21747398 PMC3223558

[13] Blest-Hopley G Giampietro V Bhattacharyya S. Residual effects of cannabis use in adolescent and adult brains - a meta-analysis of fMRI studies. Neurosci Biobehav Rev. (2018) 88:26–41. 10.1016/j.neubiorev.2018.03.008 29535069

[14] van de Giessen E Weinstein JJ Cassidy CM Haney M Dong Z Ghazzaoui R. Deficits in striatal dopamine release in cannabis dependence. Mol Psychiatry. (2017) 22:68–75. 10.1038/mp.2016.21 27001613 PMC5033654

[15] Batalla A Lorenzetti V Chye Y Yücel M Soriano-Mas C Bhattacharyya S. The influence of DAT1, COMT, and BDNF genetic polymorphisms on total and subregional hippocampal: volumes in early onset heavy cannabis users. Cannabis Cannabinoid Res. (2018) 3:1–10. 10.1089/can.2017.0021 29404409 PMC5797324

[16] Volkow ND Baler RD Compton WM Weiss SR. Adverse health effects of marijuana use. N Engl J Med. (2014) 370:2219–27. 10.1056/NEJMra1402309 24897085 PMC4827335

[17] Volkow ND Wang GJ Telang F Fowler JS Alexoff D Logan J. Decreased dopamine brain reactivity in marijuana users is associated with negative emotionality and addiction severity. Proc Natl Acad Sci USA. (2014) 111:E3149–56. 10.1073/pnas.1411228111 25024177 PMC4121778

[18] Bossong MG Mehta MA van Berckel BN Howes OD Kahn RS Stokes PR. Further human evidence for striatal dopamine release induced by administration of Δ9-tetrahydrocannabinol (THC): selectivity to limbic striatum. Psychopharmacology (Berl). (2015) 232:2723–9. 10.1007/s00213-015-3915-0 25801289 PMC4816196

[19] Rubino T Vigano D Realini N Guidali C Braida D Capurro V. Chronic delta 9-tetrahydrocannabinol during adolescence provokes sex-dependent changes in the emotional profile in adult rats: behavioral and biochemical correlates. Neuropsychopharmacology. (2008) 33:2760–71. 10.1038/sj.npp.1301664 18172430

[20] Bovasso GB. Cannabis use as a risk factor for depressive symptoms. Am J Psychiatry. (2001) 158:2033–7. 10.1176/appi.ajp.158.12.2033 11729021

[21] Chandra S Radwan MM Majumdar CG Church JC Freeman TP ElSohly MA. New trends in cannabis potency in USA and Europe during the last decade (2008–2017). Eur Arch Psychiatry Clin Neurosci. (2019) 269:5–15. 10.1007/s00406-019-00983-5 30671616

[22] Fischer AS Whitfield-Gabrieli S Roth RM Brunette MF Green AI. Impaired functional connectivity of brain reward circuitry in patients with schizophrenia and cannabis use disorder: effects of cannabis and THC. Schizophrenia Res. (2014) 158:176–82. 10.1016/j.schres.2014.04.033 25037524 PMC4778557

[23] Hunault CC Mensinga TT Böcker KB Schipper CM Kruidenier M Leenders ME. Cognitive and psychomotor effects in males after smoking a combination of tobacco and cannabis containing up to 69 mg delta-9-tetrahydrocannabinol (THC).*Psychopharmacology (Berl)*. (2009) 204:85–94. 10.1007/s00213-008-1440-0 19099294

[24] Nestor L Hester R Garavan H. Increased ventral striatal BOLD activity during non-drug reward anticipation in cannabis users. Neuroimage. (2010) 49:1133–43. 10.1016/j.neuroimage.2009.07.022 19631753 PMC2764826

[25] Liu Z Han J Jia L Maillet JC Bai G Xu L. Synaptic neurotransmission depression in ventral tegmental dopamine neurons and cannabinoid-associated addictive learning. PLoS ONE. (2010) 5:e15634. 10.1371/journal.pone.0015634 21187978 PMC3004941

[26] Blum K Febo M Smith DE Roy AK III Demetrovics Z Cronj é FJ. Neurogenetic and epigenetic correlates of adolescent predisposition to and risk for addictive behaviors as a function of prefrontal cortex dysregulation. J Child Adolesc Psychopharmacol. (2015) 25:286–92. 10.1089/cap.2014.0146 25919973 PMC4442554

[27] Blum K Liu Y Wang W Wang Y Zhang Y Oscar-Berman M. rsfMRI effects of KB220Z™ on neural pathways in reward circuitry of abstinent genotyped heroin addicts. Postgrad Med. (2015) 127:232–41. 10.1080/00325481.2015.994879 25526228 PMC4979602

[28] Blum K Noble EP Sheridan PJ Montgomery A Ritchie T. Allelic association of human dopamine D2 receptor gene in alcoholism. JAMA. (1990) 263:2055–60. 10.1001/jama.263.15.2055 1969501

[29] Blum K Chen ALC Thanos PK Febo M Demetrovics Z Dushaj K. Genetic addiction risk score (GARS) ™P, a predictor of vulnerability to opioid dependence. Front Biosci (Elite Ed). (2018) 10:175–96. 10.2741/e816 28930612

[30] Blum K Gondré-Lewis MC Baron D Thanos PK Braverman ER Neary J. Introducing precision addiction management of Reward Deficiency Syndrome, the construct that underpins all addictive behaviors. Front Psychiatry. (2018) 9:548. 10.3389/fpsyt.2018.00548 30542299 PMC6277779

[31] Blum K Lott L Siwicki D Fried L Hauser M Simpatico T. Genetic Addiction Risk Score (GARS™) as a predictor of substance use disorder: Identifying predisposition not diagnosis. Curr Trends Med Diagn Methods. (2018) 1:10.29011/CTMDM-101.100001. 10.19080/GJARM.2017.01.555556 31276118 PMC6604806

[32] Blum K Modestino EJ Gondre-Lewis M Chapman EJ Neary J Siwicki D. The benefits of Genetic Addiction Risk Score (GARS™) testing in Substance Use Disorder (SUD). Int J Genom Data Min. (2018) 1:115. 10.29011/2577-0616.000115 30198022 PMC6128289

[33] Blum K Modestino EJ Gondre-Lewis MC Baron D Thanos PK Downs BW. Pro-Dopamine Regulator (KB220) A fifty year sojourn to Combat Reward Deficiency Syndrome (RDS): evidence based bibliography (Annotated). CPQ Neurol Psychol. (2018) 1. 10.17756/jrdsas.2017-034 30957097 PMC6448775

[34] Blum K Modestino EJ Lott L Siwicki D Baron D Howeedy A. Introducing “Precision Addiction Management (PAM®)” as an adjunctive genetic guided therapy for abusable drugs in America. Open Access J Behav Sci Psychol. (2018) 1:1–4. 30662982 PMC6335959

[35] Blum K Modestino EJ Neary J Gondré-Lewis MC Siwicki D Moran M. Promoting Precision Addiction Management (PAM) to combat the global opioid crisis. Biomed J Sci Tech Res. (2018) 2:1–4. 10.26717/BJSTR.2018.02.000738 30370423 PMC6201280

[36] Blum K Siwicki D Baron D Modestino EJ Badgaiyan RD. The benefits of genetic addiction risk score (GARS™) and pro-dopamine regulation in combating suicide in the American Indian population. J Syst Integr Neurosci. (2018) 4:10.15761/JSIN.1000195. 10.15761/JSIN.1000195 31660252 PMC6816273

[37] Blum K Gold M Modestino EJ Baron D Boyett B Siwicki D. Would induction of dopamine homeostasis via coupling genetic addiction risk score (GARS®) and pro-dopamine regulation benefit benzodiazepine use disorder (BUD)? J Syst Integr Neurosci. (2018) 4:10.15761/JSIN.1000196. 10.15761/JSIN.1000196 31750006 PMC6865059

[38] Blum K Jacobs W Modestino EJ DiNubile N Baron D McLaughlin T. Insurance companies fighting the peer review empire without any validity: the case for addiction and pain modalities in the face of an American drug epidemic. SEJ Surg Pain l. (2018) 1:1–11. 29911684 PMC5998670

[39] Blum K Oscar-Berman M Blum SH Madigan MA Waite RL McLaughlin T. Can genetic testing coupled with enhanced dopaminergic activation reduce recidivism rates in the Workers Compensation Legacy Cases? J Alcohol Drug Depend. (2014) 2:161. 10.4172/2329-6488.1000161 27512720 PMC4976629

[40] Blum K Oscar-Berman M Demetrovics Z Barh D Gold MS. Genetic Addiction Risk Score (GARS): molecular neurogenetic evidence for predisposition to Reward Deficiency Syndrome (RDS). Mol Neurobiol. (2014) 50:765–96. 10.1007/s12035-014-8726-5 24878765 PMC4225054

[41] Blum K Oscar-Berman M Waite RL Braverman ER Kreuk F Li M. A multilLocus approach to treating fibromyalgia by boosting dopaminergic activity in the meso-limbic system of the brain. J Genet Syndr Gene Ther. (2014) 5:213. 10.4172/2157-7412.1000213 24883230 PMC4039556

[42] Blum K Smith DE Femino J Roy AK Simpatico T Inaba D. Hypothesizing benefits of the incorporation of genetic addiction risk (GARS_RX™_) and Dopamine Agonist Modalities (DAM) in clinical addiction medicine. J Addict Ther Res. (2014) 1:009.

[43] Downs BW Blum K Baron D Bowirrat A Lott L Brewer R. Death by opioids: Are there non-addictive scientific solutions? J Syst Integr Neurosci. (2019) 5:10.15761/JSIN.1000211. 10.15761/JSIN.1000211 31824737 PMC6904107

[44] Blum K Whitney D Fried L Febo M Waite RL Braverman ER. Hypothesizing that a pro-dopaminergic regulator (KB220z(™) liquid variant can induce “dopamine homeostasis” and provide adjunctive detoxification benefits in opiate/opioid dependence. Clin Med Rev Case Rep. (2016) 3:125. 10.23937/2378-3656/1410125 29034323 PMC5638455

[45] Blum K Febo M Fried L Baron D Braverman ER Dushaj K. Pro-dopamine regulator - (KB220) to balance brain reward circuitry in Reward Deficiency Syndrome (RDS). J Reward Defic Syndr Addict Sci. (2017) 3:3–13. 28804788 PMC5551501

[46] Blum K Febo M Fried L Li M Dushaj K Braverman ER. Hypothesizing that neuropharmacological and neuroimaging studies of glutaminergic-dopaminergic optimization complex (KB220Z) are associated with “dopamine homeostasis” in reward deficiency syndrome (RDS). Subst Use Misuse. (2017) 52:535–47. 10.1080/10826084.2016.1244551 28033474 PMC5589271

[47] Blum K Modestino EJ Gondré-Lewis MC Neary J Siwicki D Hauser M. Global opioid epidemic: Doomed to fail without genetically based addiction medicine (PAM™): lessons learned from America. Precision Med. (2017) 2:17–22. 29372187 PMC5778881

[48] Blum K Han D Hauser M Downs BW Giordano J Borsten J. Neurogenetic impairments of brain reward circuitry links to Reward Deficiency Syndrome(RDS) as evidenced by genetic addiction risk score(GARS): a case study. IIOABJ. (2013) 4:4–9.

[49] Blum K Oscar-Berman M Barh D Giordano J Gold M. Dopamine genetics and function in food and substance abuse. J Genet Syndr Gene Ther. (2013) 4:1000121. 10.4172/2157-7412.1000121 23543775 PMC3609029

[50] Blum K Oscar-Berman M Femino J Waite RL Benya L Giordano J. Withdrawal from Buprenorphine/Naloxone and maintenance with a natural dopaminergic agonist: a cautionary note. J Addict Res Ther. (2013) 4:10.4172/2155-6105.1000146. 10.4172/2155-6105.1000146 24273683 PMC3835595

[51] Blum K Giordano J Morse S Liu Y Tian J Bowirrat A. Genetic Addiction Risk Score (GARS) analysis: Exploratory development of polymorphic risk alleles in poly-drug addicted males. Integr Omics Appl Biotechnol. (2010) 1:1–14.

[52] Blum K Chen AL Oscar-Berman M Chen TJ Lubar J White N. Generational association studies of dopaminergic genes in reward deficiency syndrome (RDS) subjects: selecting appropriate phenotypes for reward dependence behaviors. Int J Environ Res Public Health. (2011) 8:4425–59. 10.3390/ijerph8124425 22408582 PMC3290972

[53] Chen TJ Blum K Chen AL Bowirrat A Downs WB Madigan MA. Neurogenetics and clinical evidence for the putative activation of the brain reward circuitry by amino-acid precursor-catabolic enzyme inhibition therapeutic agent (a Neuroadaptagen): Proposing an addiction candidate gene panel map. J Psychoactive Drugs. (2011) 43:108–27. 10.1080/02791072.2011.587393 21858957

[54] Bowirrat A Chen TJ Oscar-Berman M Madigan M Chen AL Bailey JA. Neuropsychopharmacology and neurogenetic aspects of executive functioning: should reward gene polymorphisms constitute a diagnostic tool to identify individuals at risk for impaired judgment? Mol Neurobiol. (2012) 45:298–313. 10.1007/s12035-012-8247-z 22371275 PMC3681950

[55] Blum K Giordano J Han D. Coupling the genetic addiction risk score (GARS), comprehensive analysis of reported drugs (CARD) and KB220Z showing reward circuitry activation of dopaminergic pathways with KB220Z for in treatment of Reward Deficiency Syndrome (RDS): a Paradigm Shift. Keynote Presented at International Conference on Genetic Syndromes and Gene Therapy. (2012). San Antonio, Texas.

[56] Blum K Oscar-Berman M Giordano J Downs B Simpatico T Han D Neurogenetic impairments of brain Reward circuitry links to Reward Deficiency Syndrome (RDS): potential nutrigenomic induced dopaminergic activation. J Genet Syndr Gene Ther. (2012) 3:1000e1115. 10.4172/2157-7412.1000e115 PMC3525955 23264886

[57] Blum K Oscar-Berman M Stuller E Miller D Giordano J Morse S. Neurogenetics and nutrigenomics of neuro-nutrient therapy for Reward Deficiency Syndrome (RDS): Clinical ramifications as a function of molecular neurobiological mechanisms. J Addict Res Ther. (2012) 3:139. 10.4172/2155-6105.1000139 23926462 PMC3733258

[58] Blum K Bowirrat A Baron D Lott L Ponce JV Brewer R. Biotechnical development of genetic addiction risk score (GARS) and selective evidence for inclusion of polymorphic allelic risk in substance use disorder (SUD) *J Syst Integr Neurosci*. (2021). 10.15761/JSIN.1000221 PMC7891477 33614164

[59] Fried L Modestino EJ Siwicki D Lott L Thanos PK Baron D. Hypodopaminergia and “Precision Behavioral Management” (PBM): it is a generational family affair. Curr Pharm Biotechnol. (2020) 21:528–41. 10.2174/1389201021666191210112108 31820688

[60] Febo M Blum K Badgaiyan RD Perez PD Colon-Perez LM Thanos PK. Enhanced functional connectivity and volume between cognitive and reward centers of naïve rodent brain produced by pro-dopaminergic agent KB220Z. PLoS ONE. (2017) 12:e0174774. 10.1371/journal.pone.0174774 28445527 PMC5405923

[61] Blum K Simpatico T Badgaiyan RD Demetrovics Z Fratantonio J Agan G. Coupling neurogenetics (GARS™) and a nutrigenomic based dopaminergic agonist to treat Reward Deficiency Syndrome (RDS): Targeting polymorphic reward genes for carbohydrate addiction algorithms. J Reward Defic Syndr. (2015) 1:75–80. 10.17756/jrds.2015-012 27617300 PMC5013730

[62] Blum K Briggs AH Trachtenberg MC Delallo L Wallace JE. Enkephalinase inhibition: Regulation of ethanol intake in mice. Alcohol. (1987) 4:449–56. 10.1016/0741-8329(87)90084-X 2829941

[63] Blum K Trachtenberg MC Ramsay JC. Improvement of inpatient treatment of the alcoholic as a function of neurotransmitter restoration: a pilot study. Int J Addict. (1988) 23:991–8. 10.3109/10826088809058853 2906910

[64] Blum K Trachtenberg MC Elliott CE Dingler ML Sexton RL Samuels AI. Enkephalinase inhibition and precursor amino acid loading improves inpatient treatment of alcohol and polydrug abusers: double-blind placebo-controlled study of the nutritional adjunct SAAVE. Alcohol. (1988) 5:481–93. 10.1016/0741-8329(88)90087-0 3072969

[65] Blum K Allison D Trachtenberg M Williams RW Loeblich LA. Reduction of both drug hunger and withdrawal against advice rate of cocaine abusers in a 30-day inpatient treatment program by the neuronutrient Tropamine. Curr Ther Res. (1988) 43:1204–14.

[66] Brown RJ Blum K Trachtenberg MC. Neurodynamics of relapse prevention: a neuronutrient approach to outpatient DUI offenders. J Psychoactive Drugs. (1990) 22:173–87. 10.1080/02791072.1990.10472542 2374070

[67] Blum K Trachtenberg MC Cook DW. Neuronutrient effects on weight loss in carbohydrate bingers; an open clinical trial. Curr Ther Res. (1990) 48:217–33.

[68] Cold JA. NeuRecover-SATM in the treatment of cocaine withdrawal and craving: a pilot study. ClinDrug Invest. (1996) 12:1. 10.2165/00044011-199612010-00001

[69] DeFrance JF Hymel C Trachtenberg MC Ginsberg LD Schweitzer FC Estes S. Enhancement of attention processing by Kantroll in healthy humans: a pilot study. Clin Electroencephalogr. (1997) 28:68–75. 10.1177/155005949702800204 9137870

[70] Blum K Cull JG Chen TJH Susan G-S Holder JM Wood R. Clinical evidence for effectiveness of Phencal^TM^ in maintaining weight loss in an open-label, controlled, 2-year study. Curr Ther Res. (1997) 55:10. 10.1016/S0011-393X(97)80108-7

[71] Ross J. Amino-acid precursor and enkephalinase inhibition therapy: evidence for effectiveness in treatment of “Reward Deficiency Syndrome (RDS) with particular emphasis on eating disorders. 1st Conference on Reward Deficiency Syndrome: Genetic Antecedents andClinical Pathways: 12-13 San Francisco, California. Abstracts. Mol Psychiatry (2001) 6(1 Suppl 1):S1–8. 10.1038/sj.mp.4000892 11247387

[72] Chen TJ Blum K Payte JT Schoolfield J Hopper D Stanford M. Narcotic antagonists in drug dependence: pilot study showing enhancement of compliance with SYN-10, amino-acid precursors and enkephalinase inhibition therapy. Med Hypotheses. (2004) 63:538–48. 10.1016/j.mehy.2004.02.051 15288384

[73] Blum K Chen TJ Meshkin B Downs BW Gordon CA Blum S. Reward deficiency syndrome in obesity: a preliminary cross-sectional trial with a Genotrim variant. Adv Ther. (2006) 23:1040–51. 10.1007/BF02850224 17276971

[74] Chen TJ Blum K Waite RL Meshkin B Schoolfield J Downs. Gene Narcotic Attenuation Program attenuates substance use disorder, a clinical subtype of reward deficiency syndrome. Adv Ther. (2007) 24:402–14. 10.1007/BF02849910 17565932

[75] Blum K Chen TJH Downs BW Meshkin B Blum SH Pons M. Synaptamine (SG8839), an amino-acid enkephalinase inhibition nutraceutical improves recovery of alcoholics, a Subtype of reward deficiency syndrome (RDS). Trends Appl Sci Res. (2007) 2:132–8. 10.3923/tasr.2007.132.138

[76] Blum K Chen AL Chen TJ Rhoades P Prihoda TJ Downs BW. LG839: anti-obesity effects and polymorphic gene correlates of reward deficiency syndrome. Adv Ther. (2008) 25:894–913. 10.1007/s12325-008-0093-z 18781289

[77] Blum K Chen TJH Chen ALC Rhodes P Prihoda TJ Downs BW. Dopamine D2 Receptor Taq A1 allele predicts treatment compliance of LG839 in a subset analysis of pilot study in the Netherlands. Gene Ther Mol Biol. (2008) 12:129–40.

[78] Blum K Chen TJH Williams L Chen ALC Downs WB Waite RL. A short term pilot open label study to evaluate efficacy and safety of LG839, a customized DNA directed nutraceutical in obesity: exploring nutrigenomics. Gene Ther Mol Biol. (2008) 12:371–82.

[79] Blum K Chen ALC Chen TJH Bowirrat A Waite RL Kerner M. Putative targeting of Dopamine D2 receptor function in Reward Deficiency Syndrome (RDS) by Synaptamine Complex™ Variant (KB220): Clinical trial showing anti -anxiety effects. Gene Ther Mol Biol. (2009) 13:214–30.

[80] Braverman ER Braverman D Acrui V Kerner M Downs BW Blum K. Targeting noradrenergic and dopaminergic mechanistic sites, hormonal deficiency repletion therapy and exercise: a case report. Am J Bariatric Med. (2010) 25:18–28.

[81] Miller DK Bowirrat A Manka M Miller M Stokes S Manka D. Acute intravenous synaptamine complex variant KB220™ “normalizes” neurological dysregulation in patients during protracted abstinence from alcohol and opiates as observed using quantitative electroencephalographic and genetic analysis for reward polymorphisms: part 1, pilot study with 2 case reports. Postgrad Med. (2010) 122:188–213. 10.3810/pgm.2010.11.2236 21084795

[82] Miller M Chen AL Stokes SD Silverman S Bowirrat A Manka M. Early intervention of intravenous KB220IV–neuroadaptagen amino-acid therapy (NAAT) improves behavioral outcomes in a residential addiction treatment program: a pilot study. J Psychoactive Drugs. (2012) 44:398–409. 10.1080/02791072.2012.737727 23457891 PMC4074362

[83] Chen D Liu Y He W Wang H Wang Z. Neurotransmitter-precursor-supplement intervention for detoxified heroin addicts. J Huazhong Univ Sci Technolog Med Sci. (2012) 32:422–7. 10.1007/s11596-012-0073-z 22684569

[84] McLaughlin T Oscar-Berman M Simpatico T Giordano J Jones S Barh D. Hypothesizing repetitive paraphilia behavior of a medication refractive Tourette's syndrome patient having rapid clinical attenuation with KB220Z-nutrigenomic amino-acid therapy (NAAT). J Behav Addict. (2013) 2:117–24. 10.1556/JBA.2.2013.2.8 26165932

[85] McLaughlin T Blum K Oscar-Berman M Febo M Demetrovics Z Agan G. Using the neuroadaptagen KB200z™ to ameliorate terrifying, lucid nightmares in RDS patients: the role of enhanced, brain-reward, functional CPQ neurology and psychology. J Reward Defic Syndr. (2015) 1:24–35. 10.17756/jrds.2015-006 26065033 PMC4459746

[86] Schoenthaler SJ Blum K Braverman ER Giordano J Thompson B Oscar-Berman M. NIDA-Drug Addiction Treatment Outcome Study (DATOS) relapse as a function of spirituality/religiosity. J Reward Defic Syndr. (2015) 1:36–45. 10.17756/jrds.2015-007 26052556 PMC4455957

[87] Blum K Downs BW Dushaj K Li M Braverman ER Fried L. The benefits of customized DNA directed nutrition to balance the brain reward circuitry and reduce addictive behaviors. Precis Med (Bangalore). (2016) 1:18–33. 28066828 PMC5210211

[88] McLaughlin T Febo M Badgaiyan RD Barh D Dushaj K Braverman ER. KB220Z™ a pro-dopamine regulator associated with the protracted, alleviation of terrifying lucid dreams. Can we infer neuroplasticity-induced changes in the reward circuit? J Reward Defic Syndr Addict Sci. (2017) 2:3–13. 10.17756/jrdsas.2016-022 28210713 PMC5308138

[89] McLaughlin T Han D Nicholson J Steinberg B Blum K Febo M. Improvement of long-term memory access with a pro-dopamine regulator in an elderly male: are we targeting dopamine tone? J Syst Integr Neurosci. (2017) 3:10.15761/JSIN.1000165. 10.15761/JSIN.1000165 29423319 PMC5800757

[90] Steinberg B Blum K McLaughlin T Lubar J Febo M Braverman ER. Low-Resolution Electromagnetic Tomography (LORETA) of changed brain function provoked by prodopamine regulator (KB220z) in one adult ADHD case. Open J Clin Med Case Rep. (2016) 2:1121. 27610420 PMC5012539

[91] Duquette LL Mattiace F Blum K Waite RL Boland T McLaughlin T. Neurobiology of KB220Z-glutaminergic-dopaminergic optimization complex [GDOC] as a liquid nano: clinical activation of brain in a highly functional clinician improving focus, motivation and overall sensory input following chronic intake. Clin Med Rev Case Rep. (2016) 3:104. 10.23937/2378-3656/1410104 29214221 PMC5714519

[92] Solanki N Darius P Blum K Gondre-Lewis MC A neuro-nutrient putative pro dopamine regulator mitigates alcohol intake in a rodent binge drinking model. Neuroscience Society Annual Meeting, Washington DC (2018).

[93] Steinberg B Carey E Modestino EJ Lubar J Thanos PK Baron D. Pro-dopamine regulation with KB220Z improves working memory in an adult with ADHD-A case report and replication. Open J Clin Med Case Rep. (2019) 5:1512.

[94] Manza P Tomasi D VolkowND Subcortical local functional hyperconnectivity in cannabis dependence Biol Psychiatry Cogn Neurosci Neuroimaging. (2018) 3:285–93. 10.1016/j.bpsc.2017.11.004 PMC5833305 29486870

[95] Khalsa J Baler R. Medicinal consequences of marijuana use, in ‘*Cannabis Use Disorders”*, eds: Ivan Montoya, M. D., and Susan Weiss, PhD., Springer Nature, Switzerland. (2019).

[96] National Academies of Sciences Engineering and Medicine. The Health Effects of Cannabis and Cannabinoids: The Current State of Evidence and Recommendations for Research. Washington, D. C. The National Academies Press (2017). 28182367

[97] Khalsa J. Medical and health consequences of marijuana. In: Mehamoud El Sohly, editor. Marijuana and the CannabinoidsIn:. Humana Press, Inc., New Jersey, NJ, Chapter 10 (2007). p. 237–52. 10.1007/978-1-59259-947-9_10

[98] Parnes JE Smith JK Conner BT. Reefer madness or much ado about nothing? Cannabis legalization outcomes among young adults in the United States. Int J Drug Policy. (2018) 56:116–20. 10.1016/j.drugpo.2018.03.011 29626630

[99] Everson EM Dilley JA Maher JE Mack CE. Post-Legalization opening of retail cannabis stores and adult cannabis use in Washington State, 2009-2016. Am J Public Health. (2019) 109:1294–301. 10.2105/AJPH.2019.305191 31318588 PMC6687243

[100] Kilmer B. How will cannabis legalization affect health, safety, and social equity outcomes? It largely depends on the 14 Ps. Am J Drug Alcohol Abuse. (2019) 45:664–72. 10.1080/00952990.2019.1611841 31264899 PMC9543368

[101] Jikomes N Zoorob M. The cannabinoid content of legal cannabis in washington state varies systematically across testing facilities and popular consumer products [published correction appears in Sci Rep. 10, 14406]. Sci Rep. (2020) 8:4519. 10.1038/s41598-018-22755-2 PMC5852027 29540728

[102] American Society of Addiction Medicine. ASAM Policy Statement on Marijuana, Cannabinoids and Legalization, (2015). Available online at: https://www.asam.org/Quality-Science/publications/magazine/read/article/2015/09/25/asam-issues-new-policy-statement-on-marijuana-cannabinoids-and-legalization (accessed September 18, 2020).

[103] Gray KM Carpenter MJ Baker NL DeSantis SM Kryway E Hartwell KJ. A double-blind randomized controlled trial of N-acetylcysteine in cannabis-dependentadolescents. Am J Psychiatry. (2012) 169:805–12. 10.1176/appi.ajp.2012.12010055 22706327 PMC3410961

[104] Tomko RL Jones JL Gilmore AK Brady KT Back SE Gray KM. N-acetylcysteine. A potential treatment for substance use disorders. Curr Psychiatr. (2018) 17:30–6. 30016376 PMC5993450

[105] Sidl ó Z Reggio PH Rice ME Inhibition of striatal dopamine release by CB1 receptor activation requires nonsynaptic communication involving GABA H2O2 and KATP channels. Neurochem Int. (2008) 52:80–8. 10.1016/j.neuint.2007.07.014 PMC2904528 17767979

[106] Koonin EV Altschul SF Bork P.BRCA1 protein products: functional motifs. Nat Genet. (1996) 13:266–7. 10.1038/ng0796-266 8673121

[107] Bloomfield MA Ashok AH Volkow ND Howes OD. The effects of Δ^9^-tetrahydrocannabinol on the dopamine system. Nature.(2016) 539:369–77. 10.1038/nature20153 27853201 PMC5123717

[108] Blum K Chen TJ Morse S Giordano J Chen AL Thompson J. Overcoming qEEG abnormalities and reward gene deficits during protracted abstinence in male psychostimulant and polydrug abusers utilizing putative dopamine D2 agonist therapy: part 2.*Postgrad Med*. (2010) 122:214–26. 10.3810/pgm.2010.11.2237 21084796

[109] McLaughlin T Blum K Oscar-Berman M Febo M Agan G Fratantonio JL. Putative dopamine agonist (KB220Z) attenuates lucid nightmares in PTSD patients: role of enhanced brain reward functional connectivity and homeostasis redeeming joy.*J Behav Addict*. (2015) 4:106–15. 10.1556/2006.4.2015.008 26132915 PMC4500891

[110] Mason OJ Morgan CJ Stefanovic A Curran HV. The psychotomimetic states inventory (PSI): measuring psychotic-type experiences from ketamine andcannabis. Schizophr Res. (2008) 103:138–42. 10.1016/j.schres.2008.02.020 18387788

[111] Blanco-Hinojo L Pujol J Harrison BJ Macia D Batalla A Nogue S. Attenuated frontal and sensory inputs to the basalganglia in cannabis users. Addict Biol. (2017) 22:1036–47. 10.1111/adb.12370 26934839

[112] Bloomfield MA Morgan CJ Kapur S Curran HV Howes OD. The link between dopamine function and apathy in cannabis users: an [18F]-DOPA PETimaging study. Psychopharmacology (Berl). (2014) 231:2251–9. 10.1007/s00213-014-3523-4 24696078

